# Alterations and test–retest reliability of functional connectivity network measures in cerebral small vessel disease

**DOI:** 10.1002/hbm.24967

**Published:** 2020-02-22

**Authors:** Benno Gesierich, Anil Man Tuladhar, Annemieke ter Telgte, Kim Wiegertjes, Marek J. Konieczny, Sofia Finsterwalder, Mathias Hübner, Lukas Pirpamer, Marisa Koini, Ahmed Abdulkadir, Nicolai Franzmeier, David G. Norris, José P. Marques, Peter zu Eulenburg, Michael Ewers, Reinhold Schmidt, Frank‐Erik de Leeuw, Marco Duering

**Affiliations:** ^1^ Institute for Stroke and Dementia Research (ISD) University Hospital Munich Germany; ^2^ Department of Neurology, Donders Institute for Brain, Cognition and Behaviour Radboud University Medical Center Nijmegen The Netherlands; ^3^ Department of Neurology Medical University of Graz Graz Austria; ^4^ University Hospital of Old Age Psychiatry, Universitäre Psychiatrische Dienste (UPD) Bern University of Bern Bern Switzerland; ^5^ Donders Institute for Brain, Cognition, and Behavior Radboud University Nijmegen The Netherlands; ^6^ German Center for Vertigo and Balance Disorders University Hospital Munich Germany; ^7^ Munich Cluster for Systems Neurology (SyNergy) Munich Germany

**Keywords:** cerebrovascular disease, cognition, functional brain imaging, functional networks, resting‐state fMRI, test–retest reliability

## Abstract

While structural network analysis consolidated the hypothesis of cerebral small vessel disease (SVD) being a disconnection syndrome, little is known about functional changes on the level of brain networks. In patients with genetically defined SVD (CADASIL, *n* = 41) and sporadic SVD (*n* = 46), we independently tested the hypothesis that functional networks change with SVD burden and mediate the effect of disease burden on cognitive performance, in particular slowing of processing speed. We further determined test–retest reliability of functional network measures in sporadic SVD patients participating in a high‐frequency (monthly) serial imaging study (RUN DMC—InTENse, median: 8 MRIs per participant). Functional networks for the whole brain and major subsystems (i.e., default mode network, DMN; fronto‐parietal task control network, FPCN; visual network, VN; hand somatosensory‐motor network, HSMN) were constructed based on resting‐state multi‐band functional MRI. In CADASIL, global efficiency (a graph metric capturing network integration) of the DMN was lower in patients with high disease burden (standardized beta = −.44; *p* [corrected] = .035) and mediated the negative effect of disease burden on processing speed (indirect path: std. beta = −.20, *p* = .047; direct path: std. beta = −.19, *p* = .25; total effect: std. beta = −.39, *p* = .02). The corresponding analyses in sporadic SVD showed no effect. Intraclass correlations in the high‐frequency serial MRI dataset of the sporadic SVD patients revealed poor test–retest reliability and analysis of individual variability suggested an influence of age, but not disease burden, on global efficiency. In conclusion, our results suggest that changes in functional connectivity networks mediate the effect of SVD‐related brain damage on cognitive deficits. However, limited reliability of functional network measures, possibly due to age‐related comorbidities, impedes the analysis in elderly SVD patients.

## Graphical abstract

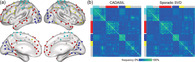

## 1 INTRODUCTION

Cerebral small vessel disease (SVD) is the leading cause of vascular cognitive impairment and dementia (Dichgans & Leys, 2017; Pantoni, 2010; Wardlaw, Smith, & Dichgans, 2019). Based on the prominent manifestation of lesions in the white matter and subcortical gray matter, SVD is commonly regarded as a disconnection syndrome and research on structural brain networks has consolidated this hypothesis (ter Telgte, van Leijsen, et al., 2018). Lower structural network integrity was associated with both higher disease burden and lower cognitive performance (Heinen et al., 2018; Lawrence, Chung, Morris, Markus, & Barrick, 2014; Reijmer et al., 2015; Tuladhar et al., 2017; Tuladhar, van Dijk, et al., 2016), depressive symptoms (Xie, Shi, & Zhang, 2017) and conversion to dementia (Lawrence, Zeestraten, et al., 2018; Tuladhar, van Uden, et al., 2016). In addition, network integrity mediated the effect of MRI‐based SVD markers on clinical symptoms (Heinen et al., 2018; Lawrence et al., 2014; Lawrence, Zeestraten, et al., 2018; Tuladhar, van Dijk, et al., 2016), underlining the significance of network disruption for expression of the clinical symptoms of the disease. However, fewer studies are available on functional connectivity in SVD (ter Telgte, van Leijsen, et al., 2018) and patient selection criteria in these studies are often rather broad, creating uncertainty as to the extent by which effects can be attributed to SVD (Fu et al., 2019; Liu et al., 2019; Nordahl et al., 2006; van Duinkerken et al., 2012; Yi et al., 2012). Furthermore, the available functional connectivity studies vary substantially in the used approach, ranging from component analysis to various seed‐based methods. In contrast, the above‐mentioned studies on structural brain networks were all based on graph theory, which might indeed be the preferred approach given its particular power and flexibility to study real‐world complex systems (Bullmore & Sporns, 2009; Rubinov & Sporns, 2010, 2011). To our knowledge, only one recent study used graph theory for the analysis of functional networks in SVD patients (Lawrence, Tozer, et al., 2018), but did not find a difference between patients and control participants. However, that study included a relatively small number of subjects and did not assess the effects of functional connectivity on cognition. Another study, which included a group of well‐defined SVD patients but did not use graph theory, found an association between functional connectivity in frontoparietal networks and cognitive performance (Cullen et al., 2016). They also conducted exploratory mediation analyses suggesting that functional connectivity might mediate the effects of structural lesions on cognitive performance.

In the current study, we addressed the hypothesis that alterations in functional networks mediate the effect of SVD burden on cognitive performance. Graph‐theory was used to characterize functional networks on the level of the global brain and major subnetworks. We included two patient samples, genetically defined SVD (cerebral autosomal dominant arteriopathy with subcortical infarcts and leukoencephalopathy; CADASIL) and sporadic SVD. CADASIL was used as a model for pure, early‐onset SVD and allowed us to largely exclude effects of age‐related comorbidities. Furthermore, the sporadic SVD patients were recruited through the RUN DMC—InTENse cohort study (ter Telgte, Wiegertjes, et al., 2018), a high‐frequency serial MR imaging study with monthly assessments over 10 months. This offered the unique opportunity to explore short‐term disease progression as well as test–retest reliability of functional network measures.

## 2 METHODS

### 2.1 Study populations

We first performed a cross‐sectional analysis in CADASIL patients, with the aim to subsequently validate results in the independent dataset of sporadic SVD patients.

The entire CADASIL sample comprised 57 patients (age < 65 years in order to minimize potential confounding by age‐related comorbidities) recruited through the VASCAMY (Vascular and Amyloid Predictors of Neurodegeneration and Cognitive Decline in Nondemented Subjects) study. Diagnosis was confirmed by either molecular genetic testing (cysteine‐altering *NOTCH3* mutation) or skin biopsy (presence of granular osmiophilic material). Five patients were excluded due to quality issues during preprocessing of structural and functional scans, and 11 patients were excluded due to excessive head motion (see below for criterion). Hence, the final CADASIL sample consisted of 41 patients.

The entire sporadic SVD sample comprised 54 patients recruited through the RUN DMC – InTENse cohort study (Radboud University Nijmegen Diffusion tensor and Magnetic resonance imaging Cohort—Investigating The origin and EvolutioN of cerebral SVD). Details of the study protocol have been published previously (ter Telgte et al., 2019; ter Telgte, Wiegertjes, et al., 2018). In short, this study included 54 patients from the previous prospective RUN DMC study with a high likelihood of progression of SVD imaging markers while meticulously excluding individuals with stroke etiologies other than SVD. All patients were scanned monthly, for 10 months. For the cross‐sectional analysis of functional networks, we used only data from the baseline MRI visit, which was closest to the neuropsychological testing session. Eight patients were excluded due to excessive head motion (see below for criterion). Hence, the final sporadic SVD sample for the cross‐sectional analysis consisted of 46 patients. The longitudinally acquired data of the same patients were used for test–retest analysis. Only patients with at least three time‐points were included in this analysis, resulting in 44 patients. These patients had a median of 8 MRI acquisitions (range from 3 to 9).

All study protocols were approved by local ethics committees of the respective institutions. Written informed consent was obtained from all subjects. Apart from covering travel costs, subjects were not compensated for their participation in the study.

### 2.2 Neuropsychological testing

The neuropsychological examination was performed on the previous or the same day as the MRI examination in the CADASIL patients (VASCAMY) and between 2 and 17 weeks (median = 8.4 weeks; IQR = 3.4 weeks) before the MRI visit in the sporadic SVD patients (RUN DMC – InTENse).

Mini‐Mental State Examination (MMSE) was used to capture the general cognitive performance in the two study samples. In order to limit the number of statistical tests, we prespecified to focus on the main cognitive deficit in SVD patients, that is, processing speed and executive function. For this purpose, we analyzed the time needed to complete the Trail Making Test matrix B (TMT‐B), which has been shown to be very sensitive toward SVD‐related deficits in these cognitive domains (Duering et al., 2011). Raw test scores were transformed into age‐ and education‐corrected *z* scores based on values from healthy subjects (Tombaugh, 2004).

### 2.3 MRI acquisition

MRI scans were performed on 3 Tesla scanners using harmonized protocols (CADASIL: Magnetom Skyra with a 64‐channel head coil; sporadic SVD: Magnetom Prisma with 32‐channel head coil; Siemens Healthineers, Erlangen, Germany). Protocols in both studies included 3D‐T1, 3D fluid‐attenuated inversion recovery (FLAIR), fast low angle shot (FLASH, T2*‐weighted), a multi‐shell diffusion‐weighted imaging sequence, together with *a b* = 0 image with inverted phase encoding direction for correcting susceptibility induced distortions, and a resting‐state multi‐band echo planar imaging pulse sequence (TR/TE 700/39 ms, flip angle 52°, multi‐band acceleration factor 8, number of volumes 675 [CADASIL] or 700 [sporadic SVD], in‐plane resolution and slice thickness 3 mm [CADASIL] or 2.4 mm [sporadic SVD]) together with two spin‐echo echo planar images with opposing phase encoding directions for field map calculation. Complete acquisition parameters are listed in Table S1. All scans from both study samples underwent rigorous visual quality control by B.G., M.D., and M.H.

### 2.4 Conventional SVD imaging markers

White matter hyperintensity (WMH) volume, number and volume of lacunes, and number of cerebral microbleeds were assessed according to consensus criteria (Wardlaw et al., 2013). In the VASCAMY dataset (CADASIL patients), WMH were segmented from registered and bias‐corrected T1 and FLAIR images, using a variant of the 3D U‐Net (Long, Shelhamer, & Darrell, 2015; Ronneberger, Fischer, & Brox, 2015) deep learning algorithm. This algorithm was trained and validated in an independent sample of 117 patients with CADASIL from a previous, prospective study (Duering et al., 2013). The WMH segmentations were then manually edited and cleaned from misclassified artifacts using a custom 3D editing tool, written in MATLAB (R2016b, The MathWorks, Natick, MA). Lacunes were detected manually on T1 images, and segmented using seed‐growing with a manually selected intensity threshold. Microbleeds were visually rated on T2*‐weighted images. In the RUN DMC—InTENse dataset (sporadic SVD patients), these markers were assessed as previously described (ter Telgte et al., 2019). All volumes were normalized to the total intracranial volume, which was approximated from tissue probability maps produced using the Statistical Parametric Mapping (SPM) toolbox tissue segmentation algorithm (v12; Wellcome Department of Cognitive Neurology, London, UK; http://www.fil.ion.ucl.ac.uk/spm). The tissue maps for gray matter, white matter, and cerebrospinal fluid were thresholded and combined in order to obtain the total intracranial volume. Brain volume was estimated as the combined volume of the thresholded gray and white matter tissue maps, and normalized for by dividing through total intracranial volume.

### 2.5 Diffusion imaging based SVD burden marker

Diffusion tensor imaging is the gold‐standard method to assess disease burden in SVD. We have previously shown that diffusion tensor imaging alterations in SVD, such as an increase in mean diffusivity or decrease in fractional anisotropy, are largely driven by an increased extracellular free water (FW) content (Duering et al., 2018). Thus, it is sufficient to determine the FW content in order to fully capture diffusion tensor imaging alterations in SVD. The FW measure ranges from 0 to 1 and corresponds to the relative content of freely diffusing, extracellular water in the observed volume (i.e., each voxel). Before calculating FW, we preprocessed diffusion data in order to reduce the effect of spatially varying noise (Veraart, Fieremans, et al., 2016; Veraart, Novikov, et al., 2016), Gibbs ringing artifacts (Kellner, Dhital, Kiselev, & Reisert, 2016), susceptibility‐induced distortions (J. L. Andersson, Skare, & Ashburner, 2003), and eddy current‐induced distortions and head motion (J. L. R. Andersson & Sotiropoulos, 2016). This was done using tools from MRtrix3 (http://www.mrtrix.org/, dwidenoise, mrdegibbs) and the Functional Magnetic Resonance Imaging of the Brain (FMRIB) Software Library (FSL; version 5.0.9, eddy, topup) (Smith et al., 2004). After preprocessing, fractional anisotropy and FW were calculated as previously described (Duering et al., 2018). Briefly, diffusion tensors were estimated using linear least squares implemented in MATLAB. FW was estimated using a nonlinear regularized minimization process (Pasternak, Sochen, Gur, Intrator, & Assaf, 2009). Using the Tract‐Based Spatial Statistics pipeline from FSL, the fractional anisotropy images were registered nonlinearly to the FMRIB58 standard space image and projected onto a custom white matter skeleton (Baykara et al., 2016). The nonlinear warps and skeleton projections from this processing step were then applied to the FW images, in order to project voxels from the native diffusion image space onto the standard space white matter skeleton template. Finally, the hereby produced skeletonized FW images were averaged, resulting in a global score for FW in the main white matter tracts. This global score was used throughout this study as a measure for the SVD burden and, for simplicity, will be referred to hereafter as FW.

### 2.6 Resting‐state functional MRI processing

Processing of resting‐state functional MRI data was done with a particular effort to account for the heavily lesioned SVD brains during spatial normalization and to minimize head motion effects. The first 10 EPI volumes were discarded, to allow the magnetization to stabilize to a steady state. The remaining volumes were realigned and corrected for static and dynamic distortions in a single step, using the “realign & unwarp” function in SPM together with a field map, calculated with FSL topup from spin‐echo EPI images with opposite phase encoding directions. Images were then normalized to Montreal Neurological Institute (MNI) space using the Advanced Normalization Tools (ANTs, version 2.1.0, [Avants et al., 2011]) and a bimodal custom template (consisting of T1 and FLAIR), created with ANTs from an independent set of 15 healthy (mean age 70 years) and 15 CADASIL patients (mean age 51 years) acquired during a previous study (Baykara et al., 2016). For each patient, the mean EPI volume was registered to the T1 image. The T1 and FLAIR images were (co‐)registered nonlinearly to the bimodal custom template, which again was normalized into MNI space. WMH and lacune lesion masks were provided to the normalization algorithm to avoid image distortions, typically resulting during nonlinear normalization of lesioned brains. Each EPI volume was transformed into MNI space by applying the combined transformations. EPI volumes in standard space were spatially smoothed (6 mm FWHM) and independent component analysis (ICA)‐AROMA (Pruim, Mennes, Buitelaar, et al., 2015; Pruim, Mennes, van Rooij, et al., 2015) was applied, using the nonaggressive denoising strategy. ICA‐AROMA is a strategy based on ICA to further reduce motion artifacts in fMRI data. Finally, nuisance regression (6 motion parameters and signal time‐courses from regions in the white matter, the cerebrospinal fluid and the whole brain), linear trend removal, band‐pass filtering (0.01–0.08 Hz), and motion scrubbing was conducted. Motion scrubbing followed a previously established protocol (Power et al., 2014; Power, Barnes, Snyder, Schlaggar, & Petersen, 2012) based on framewise displacement, calculated using custom MATLAB code (BRAMILA pipeline v2.0, available at https://git.becs.aalto.fi/bml/bramila/). Volumes that exhibited a framewise displacement >0.5 mm were removed together with one preceding and two subsequent volumes. Participants were excluded if the acquisition time, after removing censored volumes, amounted to less than 5 min. We chose this threshold, because it has been previously shown that estimates of correlation strength stabilize with acquisition times as brief as 5 min (Van Dijk et al., 2010). As mentioned above, this applied to 11 CADASIL, 8 sporadic SVD patients and 60 out of 378 visits from the 44 sporadic SVD patients in the longitudinal dataset. As consequence of motion scrubbing, the median number (and the first and third quartile) of remaining volumes was 653 (608, 665) in the 41 CADASIL patients, 674 (643, 686) in the 46 sporadic SVD patients, and 674 (641, 686) in the longitudinal dataset.

### 2.7 Network reconstruction and graph analysis

**Figure 1:**
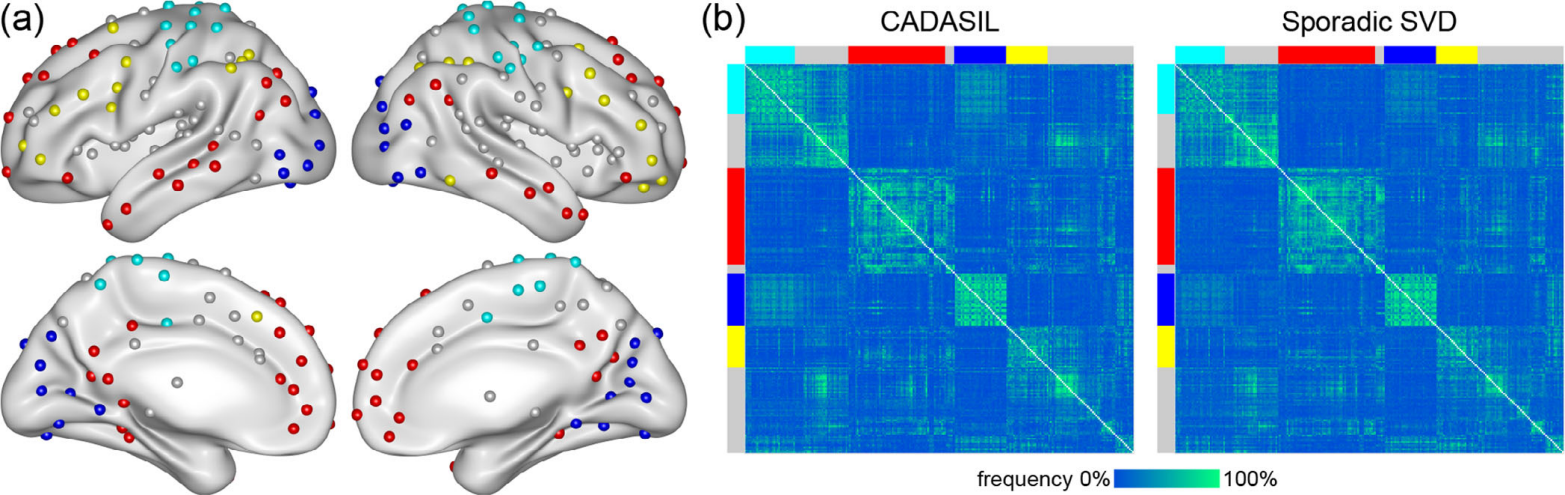
Network reconstruction. (a) Functional areas used as network nodes: the default mode network (DMN; red), fronto‐parietal task control network (FPCN; yellow), visual network (VN; blue), and hand somatosensory‐motor network (HSMN; cyan). For reconstruction of the global network, nodes of other functional systems (gray) were included as well. (b) Percentage of patients for whom an edge was included in the graph‐analysis, using the 20% density threshold. Functional systems are indicated by colors as in Panel a

Functional brain networks were represented using graph theory. A graph is defined as a mathematical object consisting of a set of items, called nodes, and pairwise relationships between them, called edges. As nodes we used a set of 264 putative functional areas (10 mm diameter spheres), defined by Power et al. (2011), which spans the cerebral cortex, subcortical structures and cerebellum. The authors also suggested a partitioning of their set of 264 areas into 12 nonoverlapping functional systems. Based on this partitioning we defined five networks: A global network of the brain was based on the 232 areas assigned to any of the 12 functional systems (32 nodes were not assigned to any network). The other four networks were based on the areas of the four largest functional systems: the default mode network (DMN, containing 58 areas), the fronto‐parietal task control network (FPCN; 25 areas), the somatosensory‐motor network of the hand (HSMN; 30 areas), and the visual network (VN; 31 areas). All other functional systems of the partitioning contained less than 20 areas and were not analyzed as separate networks, following the recommendation of the Brain Connectivity Toolbox for a minimum number of 20 network nodes in order to conduct a meaningful graph theoretical analysis. The nodes of the global and the four selected functional systems are shown in Figure 1a. The edges of these networks were defined as the functional connectivity between all pairs of nodes, calculated as the temporal correlation (Pearson's *r*) in the blood‐oxygen‐level‐dependent (BOLD) signal between nodes. Hence, for a network with *N* nodes, the entirety of all edges was defined as a *N* × *N* correlation matrix. One such correlation matrix (connectivity matrix) was calculated for each of the five networks and for each participant. The diagonal of each matrix was set to zero. A critical step of graph analysis is thresholding of the correlation matrices to remove spurious connections and to obtain sparsely connected matrices (van den Heuvel et al., 2017; van Wijk, Stam, & Daffertshofer, 2010). We used proportional thresholding, which keeps the network density constant across subjects, because network density has a direct effect on many graph metrics (van Wijk et al., 2010). Proportional thresholding is conducted by keeping the same, predefined number of strongest edges for all subjects. However, proportional thresholding may also lead to modified network topologies by enforcing nonsignificant connections or ignoring significant connections, depending on differences across subjects in overall functional connectivity (van Wijk et al., 2010). To account for this possible pitfall, we tested whether SVD burden affected overall functional connectivity, which we calculated by averaging across all positive valued edges in the network matrix as suggested by van den Heuvel et al. (2017). To evaluate robustness of findings, networks were thresholded repeatedly for a range of 9 density thresholds from 5 to 45%, with a step‐size of 5%. Densities higher than 45% were not analyzed, since this would have caused inclusion of negative valued edges. For illustration, Figure 1b shows the edge‐wise percentage of patients for whom an edge was included in the graph analysis when using the 20% density threshold.

Graph theoretic measures were calculated for each of the five functional networks, for each of the nine density thresholds and for each participant, using the Brain Connectivity Toolbox (Rubinov & Sporns, 2010). We a priori selected two measures: the weighted global efficiency (GE), reflecting functional integration in the network, and the weighted clustering coefficient (CC), reflecting functional segregation in the network. These two graph theoretic measures (GE and CC) are commonly used and were previously reported to be sensitive also to structural network changes in SVD (Lawrence, Tozer, et al., 2018). There were strong correlations between the two network measures at less stringent thresholds (25% density and above), but not at the more stringent thresholds (Figure S1).

### 2.8 Statistical analysis

All statistical analyses were performed in R (version 3.6.0; [R Core Team, 2016]). Sample characteristics were compared between the CADASIL and sporadic SVD samples using Chi‐squared test (for categorical variables) or Wilcoxon rank sum test (for numerical variables). For all other analyses, variables were first power‐transformed using the Yeo‐Johnson transformation (Yeo & Johnson, 2000), as implemented in the R package “car” (version 3.0‐0; (J. Fox & Weisberg, 2011)), to approximate normal distribution.

For the cross‐sectional analysis, we first tested for simple pairwise associations between disease burden (i.e., FW), network measures (i.e., GE and CC calculated for the global network, FPCN, DMN, HSMN, and VN) and processing speed (i.e., TMT‐B *z*‐scores), using simple linear regression models. These analyses were repeated for all density thresholds. We considered the number of multiple comparisons for these simple regression models to be *n* = 10, resulting from multiplying the numbers of analyzed networks (*n* = 5) and network measures (*n* = 2). The repetition of analyses for the range of density thresholds (*n* = 9) was not considered for multiple comparison correction, given strong dependencies of network measures across thresholds. Correction was performed using the Bonferroni method.

In case of significant pairwise associations between the three types of variables (i.e., disease burden, network measure, processing speed), a formal mediation analysis was performed using path analysis as implemented in the R package “lavaan” (version 0.6‐3; (Rosseel, 2012)), with processing speed as dependent variable, disease burden as predictor and the respective network measure as mediator. *P*‐values were estimated using bootstrap analysis (50,000 repetitions).

Longitudinal analysis was performed in sporadic SVD patients (RUN DMC—InTENse study) to assess changes in disease burden (i.e., FW) and functional network measures (i.e., GE, CC) over time. We estimated linear mixed models with the R package “lme4” (version 1.1‐21; [Bates, Mächler, Bolker, & Walker, 2014]), and calculated *p*‐values with the R package “lmerTest” (version 3.1.0; (Kuznetsova, Brockhoff, & Christensen, 2017)). Time of MRI visits (relative to baseline visit) was modeled as fixed effect, including a random intercept and slope for each subject. Test–retest reliability of disease burden and functional network measures was estimated using intraclass correlation coefficient (ICC) with the R package “psych” (1.8.12; (Revelle, 2019)) and applying the one‐way ANOVA model, that is, ICC(1,1) (Shrout & Fleiss, 1979). The ICC is the ratio of variance due to subjects (intersubject variance) to the total variance (sum of intersubject and intrasubject/error variance) and is a measure of how correlated (similar) measurements are within the same person. According to commonly used guidelines for clinical research (Fleiss, 1986), ICC results were considered to be “excellent” (ICC >0.75), “fair to good” (0.40–0.75) or “poor” (<0.40).

We determined the influence of multiple variables (i.e., age, sex, mean framewise displacement as a summary score for head motion, FW, brain volume, WMH volume, hypertension, diabetes, hypercholesterolemia, smoking) on the variability of network measures across visits. For this purpose, we first calculated the standard deviation of both network measures (GE and CC) across visits for each subject. These standard deviations (one per subject) were then regressed against the above listed explanatory variables. Similarly, as in the context of the cross‐sectional analyses described above, we considered the number of multiple comparisons done for each explanatory variable to amount to *n* = 10, and corrected using Bonferroni method.

## 3 RESULTS

### 3.1 Demographics, cognition and disease burden

The demographic, clinical and radiological characteristics of CADASIL and sporadic SVD patients are depicted in Table 1. A direct comparison between groups was not intended by this study and the differences in age and disease burden are consequences of the study design (intentionally including young CADASIL patients to avoid age‐related comorbidities).

**Table 1:** Characteristics of the samples at baseline visit

Variable	CADASIL (VASCAMY)	Sporadic SVD (RUN DMC–InTENse)	*p*
*n*	41	46	
*Demographic characteristics*			
Age, years	54 (14.8) [32,64]	68 (8.4) [61,90]	<.001
Female, *n* (%)	27 (65.9)	18 (39.1)	.013
Education, years	10 (3) [8,20]	11 (4) [8,18]	.180
*Vascular risk factors*			
Smoking (current and past), *n* (%)	27 (65.9)	31 (67.4)	.879
Hypertension, *n* (%)	11 (26.8)	37 (80.4)	<.001
Hypercholesterolemia, *n* (%)	18 (43.9)	21 (45.7)	.870
Diabetes, *n* (%)	0 (0)	6 (13.0)	.017
*Neuropsychology*			
TMT‐B, *Z*‐score	−0.4 (2.1) [−9.2,1.5]	0 (1.8) [−9.0,1.6]	.322
MMSE score	29 (2) [23,30]	29 (2) [26,30]	.252
*MRI SVD markers*			
FW	0.28 (0.09) [0.18,0.56]	0.17 (0.04) [0.14,0.31]	<.001
WMH volume, %	4.1 (5) [0.08,15.1]	0.3 (0.6) [0.03,3.1]	<.001
Lacune volume, %	0 (0) [0,0.08]	0 (0) [0,0.05]	<.001
Lacune count	2 (3) [0,21]	0 (0) [0,20]	<.001
Brain volume, %	76.0 (7.2) [66.9,86.2]	77.3 (5.5) [64.4,85.8]	.131
Cerebral microbleeds count	2 (6) [0,21]	0 (1) [0,9]	<.001

*Note*: Lesion and brain volumes are normalized by the total intracranial volume. For numeric variables median (interquartile range) [min, max] is shown.

Abbreviations: FW, free water content; MMSE, Mini‐Mental State Examination; SVD, small vessel disease; TMT‐B, Trail Making Test matrix B, age and education adjusted *z*‐scores; WMH, white matter hyperintensity.

A comparable association between disease burden (measured by FW) and processing speed (measured by TMT‐B) could be observed in CADASIL (standardized beta = −.39; adjusted *R* ^*2*^ = 13.3%; *p* = .011) and sporadic SVD patients (standardized beta = −.36; adjusted *R* ^*2*^ = 11.2%; *p* = .013), confirming suitability of FW as a measure for SVD burden in both patient groups.

### 3.2 Cross‐sectional functional network analysis

**Figure 2:**
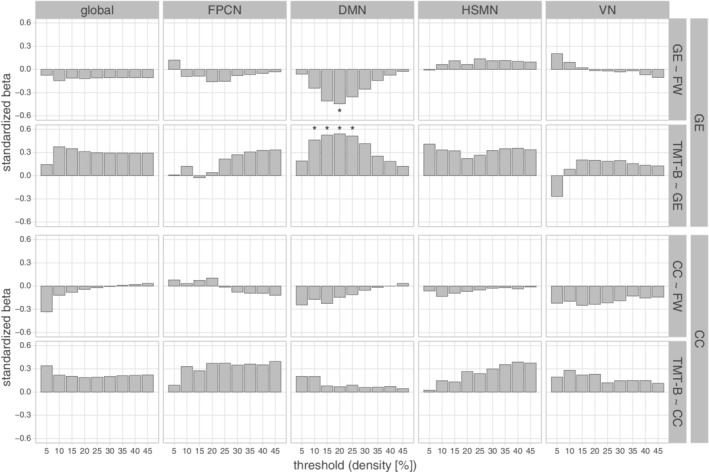
Cross‐sectional analysis in CADASIL using simple regression. Standardized beta estimates are shown for regression of network measures against disease burden (GE ~ FW; CC ~ FW) and processing speed against network measures (TMT‐B ~ GE; TMT‐B ~ CC). Analyses were performed for the five reconstructed networks (columns) using different density thresholds (*x*‐axis). * *p* < .05 (Bonferroni corrected). Abbreviations: CC, weighted clustering coefficient; DMN, default mode network; FPCN, fronto‐parietal task control network; FW, free water content within main white matter tracts; GE, weighted global efficiency; global, global network; HSMN, hand somatosensory‐motor network; TMT‐B, Trail Making Test matrix B; VN, visual network

**Figure 3:**
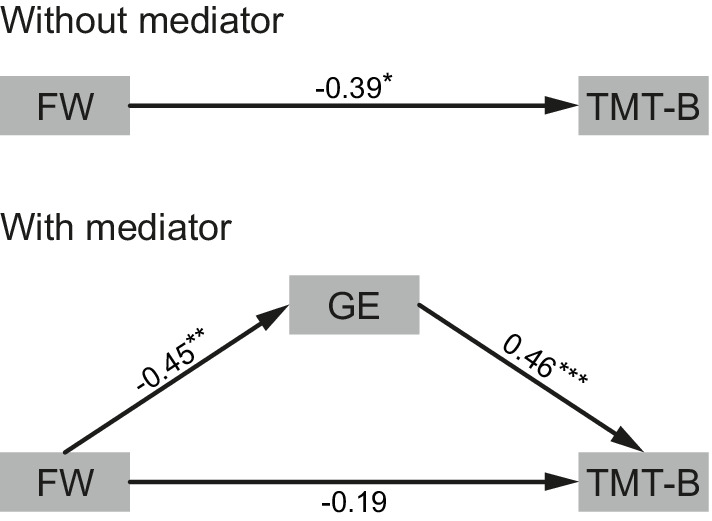
Mediation analysis in CADASIL patients. Weighted global efficiency (GE) in the default mode network mediates the effect of disease burden (FW) on processing speed (TMT‐B). The model is shown without (top) and with (bottom) mediator. Standardized beta estimates are shown for each path (*p < .05; **p < .01; ***p < .001)

We tested associations of network measures (i.e., GE and CC) with disease burden (i.e., FW) as well as with processing speed (i.e., TMT‐B), using simple regression analysis. In CADASIL, there was a significant association between GE and FW. This association was specifically present in the DMN (20% density threshold, adjusted *R* ^*2*^ = 17.7%, corrected *p* = .036; Figure 2). Importantly, a significant association was also found between GE and TMT‐B, again only in the DMN (density thresholds from 10 to 25%, maximum adjusted *R* ^*2*^ = 27.5% and minimum corrected *p* = .0026 at density 20%; Figure 2). No significant associations were found in the sporadic SVD patients (Figure S2). Therefore, mediation analysis was only conducted in CADASIL, and only with GE in the DMN (calculated at 20% density threshold) as mediator. The effect of disease burden on processing speed was mediated by GE in the DMN (indirect path: standardized beta = −.20, *p* = .047; direct path: standardized beta = −.19, *p* = .25; total effect: standardized beta = −.39, *p* = .02; Figure 3).

Regressing overall functional connectivity in the DMN of CADASIL patients against FW (uncorrected *p* = .74) confirmed that the association between disease burden and the graph‐theoretical network measure GE was not indirectly driven by a disease burden associated difference in overall functional connectivity (van den Heuvel et al., 2017). There was also no effect of disease burden on overall functional connectivity in any other network, neither in CADASIL (smallest uncorrected *p* = .32, in the visual network) nor in the sporadic SVD sample (smallest uncorrected *p* = .11, in the global network).

### 3.3 Longitudinal analysis and test–retest reliability in sporadic SVD

**Figure 4:**
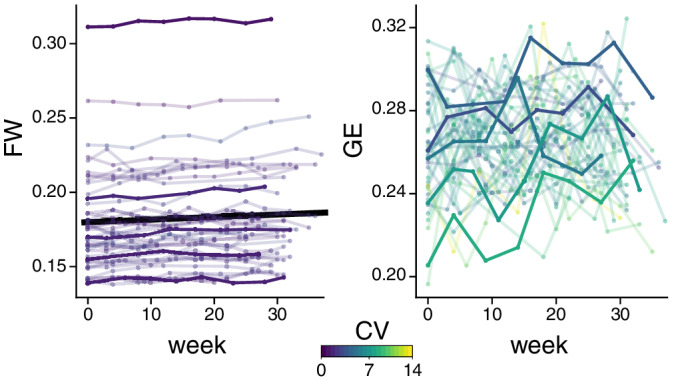
Longitudinal analysis of the monthly MRI data in the sporadic SVD sample. Free water (FW, left) and global efficiency of the default mode network (at 20% density threshold, GE, right) versus time. Lines correspond to individual subjects, color‐coded by their coefficient of variation (CV). To allow better appreciation of single subject time‐courses, five subjects (with equally spaced mean values) are highlighted. Only FW increased significantly over time, as indicated by the black line

Disease burden, as measured by FW, increased over time (median 31 weeks follow‐up) in the sporadic SVD sample (linear mixed model, beta = .00017, *p* = 1.8e − 08, Figure 4a). In contrast, network measures (GE and CC) did not change over time for any threshold or network (range of uncorrected *p*‐values .059–.999, Figure 4b).

**Figure 5:**
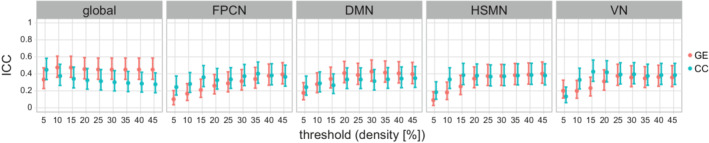
Test–retest reliability of network measures in the sporadic SVD sample. The intraclass correlation (ICC) coefficient is shown for all networks and for different density thresholds (*x*‐axis). Point estimates and 95% confidence interval are shown. Abbreviations: CC, weighted clustering coefficient; DMN, default mode network; FPCN, fronto‐parietal task control network; GE, weighted global efficiency; global, global network; HSMN, hand somatosensory‐motor network; VN, visual network

Test–retest reliability was assessed in the longitudinal dataset for the network measures GE and CC, calculating ICC at all density thresholds and for all networks. The ICC(1,1) was in all cases below or marginally above 0.4 (GE: median = 0.364; min/max = 0.091/0.475; CC: median = 0.344; min/max = 0.134/0.448; Figure 5), indicating poor to fair reliability. In comparison, FW showed excellent reliability (ICC(1,1) = 0.988; 95% confidence interval = [0.982,0.992]).

Finally, we explored factors explaining differences across subjects, regarding the variability of network measures across visits. We tested the effect of age, sex, head‐motion (mean framewise displacement), SVD markers (FW, brain volume, WMH volume), and vascular risk factors (hypertension, diabetes, hypercholesterolemia, smoking). The variability across visits of GE in the DMN was higher in older patients (maximum standardized beta = .58 and minimum corrected *p* = 4.1e − 04 at density 35%, Figure S3). Similar, but only marginally significant effects of age on network measure variability were also found for CC in the DMN (standardized beta = .41 and corrected *p* = .061 at density 20%) and for GE in the global network (standardized beta = .40 and corrected *p* = .07 at density 25%). No significant effects were found for the other variables.

## 4 DISCUSSION

We found lower functional integration in the DMN (as measured by global efficiency) in patients with higher disease burden in the genetically defined SVD study group. This functional disintegration in the DMN mediated the effect of disease burden on processing speed, the cognitive domain predominantly affected in SVD. While these findings in CADASIL patients support the view of SVD being a disconnection syndrome, we were not able to independently validate these results in sporadic SVD patients. A plausible explanation is the poor test–retest reliability of network metrics in sporadic SVD patients, as revealed by the analysis of high‐frequency serial imaging data. Age was the only factor associated with increased variability across visits, suggesting that age‐related comorbidities other than SVD might underlie the poor reliability of functional network measures.

### 4.1 Functional network properties mediate the effect of disease burden

In CADASIL patients, the effect of disease burden on cognition was mediated by decreased network efficiency in the DMN. While we can only speculate on why disease burden was specifically associated with alterations in this network, there is supporting evidence from another study in CADASIL (Cullen et al., 2016). That study used ICA to define networks and analyzed mean connectivity in four of the resulting components, which the authors labeled as attentional or executive. Importantly, these networks showed considerable overlap with areas typically considered part of the DMN. In particular, the strongest correlations with processing speed as well as executive function were found by Cullen et al. for one component dominated by a large cluster in the bilateral precuneus, an area typically implicated in the DMN. When comparing to the atlas used in our study (Power et al., 2011), the cluster is indeed in vicinity to a ROI assigned to the DMN.

The DMN is involved in a wide variety of cognitive functions, such as episodic memory, envisioning the future, mentalizing and self‐referential mental activity (Buckner, Andrews‐Hanna, & Schacter, 2008; Davey, Pujol, & Harrison, 2016; K. C. Fox, Spreng, Ellamil, Andrews‐Hanna, & Christoff, 2015; Raichle, 2015; Spreng, Mar, & Kim, 2009). One hypothesis suggests that the DMN supports all these different cognitive activities by allowing cognition to uncouple from the actual perceptual experience and to be shaped by information from stored representations (Konishi, McLaren, Engen, & Smallwood, 2015; Murphy et al., 2018; Smallwood et al., 2013; Spreng et al., 2014). The TMT‐B used in our study was designed to probe processing speed and executive functioning. However, the test also relies strongly on the ability to guide cognition by memory rather than sensory input alone. In order to identify the next target symbol, the preceding symbol has to be remembered. Compared to the complex cognitive activities predominantly associated with the DMN, such as envisioning the future or mentalizing, the TMT‐B is a simple task. However, the involvement of the DMN in rather simple cognitive functions has been confirmed by task fMRI (Konishi et al., 2015), thus making a role of the DMN for processing speed performance in SVD patients plausible.

### 4.2 Test–retest reliability

The value of a measure depends strongly on its precision relative to its normative intersubject variation, which can be assessed by ICC. In the group of sporadic SVD patients, we had the unique opportunity to assess reliability of imaging measures using multiple timepoints (median = 8) from a monthly serial imaging study. Across all tested networks and thresholds, we found poor test–retest reliability of functional network measures, with most ICC values below 0.4. To our knowledge there is only one study investigating test–retest reliability of graph based functional network metrics in SVD patients, which found ICC values close to zero (Lawrence, Tozer, et al., 2018). The lower ICC values in that study could be due to methodological differences, which have been shown to strongly influence reliability of graph analysis of functional network metrics (Andellini, Cannata, Gazzellini, Bernardi, & Napolitano, 2015; Termenon, Jaillard, Delon‐Martin, & Achard, 2016).

We further explored determinants of high within‐subject variability of network metrics and found higher variability with older age, but not with increased SVD burden. To our knowledge, only few studies investigated factors impacting on reliability of functional network metrics in normal and pathological aging. Seemingly in contrast with our study, which found no effect of SVD burden, the above‐mentioned study by Lawrence and colleagues found lower reliability in SVD patients compared with healthy controls. Since all of our subjects had SVD, we cannot exclude that the presence of SVD has an effect on reliability. However, our data suggests that the degree of SVD severity plays a minor role. Another study compared young and elderly healthy subjects and found that age reduced the reliability of functional connectivity, calculated as correlation coefficients between BOLD signal time series in 92 regions of interest (Song et al., 2012). Although not investigated by these authors, a likewise reduced reliability of derived graph metrics seems very plausible. A further study found test–retest reliability of graph based functional network metrics in older adults to be low (Guo et al., 2012), but did not explore the effect of age or age‐related pathologies. A study, comparing subjects with mild cognitive impairment to healthy seniors, found a reduced test–retest reliability of ICA‐derived resting‐state networks (Blautzik et al., 2013). In summary, our findings suggest that aging and age‐related pathologies other than SVD severity contribute to decreased reliability of graph based functional network metrics in elderly subjects.

### 4.3 Missing generalization to sporadic SVD

Results in the sporadic SVD sample were negative and, hence, we were not able to generalize our findings from genetically defined to sporadic SVD. Although previous studies in SVD differed in terms of methodology, they support the notion that the investigation of functional connectivity is more challenging in sporadic SVD compared with genetically defined SVD. While a previous study in CADASIL patients linked resting‐state connectivity to cognitive performance (Cullen et al., 2016), a study in sporadic SVD did not find differences in function connectivity between patients and healthy controls (Lawrence, Tozer, et al., 2018).

The findings on test–retest reliability provide a plausible explanation why the mediation effect was only found in the relatively young CADASIL patients. Age and age‐related comorbidities might have reduced reliability of the network metrics in the older sporadic SVD patients, thus reducing statistical power. However, as we could not assess test–retest reliability in our CADASIL sample, this conclusion remains speculative.

Age‐related comorbidities might not only reduce reliability, but exert their own effects on network metrics. In this way, even subclinical comorbidities might introduce noise and potentially mask the SVD‐related effect on network metrics. Another explanation might be the difference in SVD burden, as reflected by the MRI markers (see Table 1). The higher and more variable SVD burden in CADASIL patients might have provided more statistical power to explore associations with functional connectivity.

### 4.4 Strengths and limitations

The current study has several strengths. First, we used two independent study samples, genetically defined SVD (CADASIL) and sporadic SVD. This allowed us to study functional network changes first in a pure form of SVD, minimizing the confounding of results by other age‐related changes and comorbidities, which indeed might have hampered the analysis in the sporadic SVD group. A further strength is the relatively large number of participants in each study sample, which is high compared with previous studies on functional connectivity in SVD (Cullen et al., 2016; Lawrence, Tozer, et al., 2018). Also, the state‐of‐the‐art spatial normalization of the data into MNI space constitutes an important strength. Structural images are heavily affected by SVD‐related lesions and normalization with standard procedures can lead to highly inaccurate results (Duering et al., 2011). We accounted for this difficulty, using a custom template, lesion masking and an advanced normalization algorithm.

Finally, a unique strength of our study is the availability of high‐frequency, serial MRI in 44 sporadic SVD patients, which enabled us to assess test–retest reliability and explore sources of variability based on both a large sample size and a high number of tests per subject (in total 319 MRI scans).

Our study also has limitations. The effect of disease burden on functional network integrity in CADASIL is based on cross‐sectional data only. Hence, we cannot infer how functional connectivity changes over the course of the disease in individual patients. Longitudinal studies with long‐term follow‐ups would be needed to address these questions. In addition, with resting‐state data alone, we cannot investigate how functional connectivity during rest relates to activity in the DMN during cognitive tasks. Future studies using task fMRI might shed more light on the role of the DMN in SVD‐related cognitive impairment. A further limitation concerns the longitudinal analysis. Measuring network metrics over time is challenging, because the thresholding of the connectivity matrices might cause a slightly different set of edges to be included at each time point. In addition, the lack of a healthy control group precludes to study the effect of SVD on functional network reliability in comparison to healthy elderly. Finally, we did not collect data on neurovascular coupling, and therefore cannot assess the extent to which disease related changes in neurovascular coupling, rather than changes in neuronal activity, underlie our results. Changes in neurovascular coupling might cause delayed or decreased BOLD responses to neural activity (Dumas et al., 2012; Huneau et al., 2018; Iadecola, 2017), which could have contributed to the reduced test–retest reliability and negative result in the sporadic SVD sample. On the other hand, we regard it as unlikely that BOLD changes caused predominantly by changes in neurovascular coupling (rather than by changes in neuronal activity) result in an association between functional‐connectivity‐based network measures and cognition.

## 5 CONCLUSION

Our study corroborates the concept of SVD being a disconnection syndrome, with altered functional network integrity mediating the effect of subcortical SVD burden on the typical cognitive deficits. While these effects were present in the genetically defined CADASIL patients, they could not be generalized to sporadic SVD. A plausible explanation for the lack of findings in the sporadic SVD sample is the reduced reliability of functional network metrics with higher age, as demonstrated by our serial imaging study. This finding of low reliability in the older, sporadic SVD patients also has implications beyond SVD, highlighting the challenges of applying graph‐based measures of functional network properties in studying populations of elderly people.

## Supporting information


**Appendix** **S1:** Supporting information

## Data Availability

The data that support the findings of this study are available on request from the corresponding author. The data are not publicly available due to privacy or ethical restrictions.
