# Supplementary material for: Alterations and test–retest reliability of functional connectivity network measures in cerebral small vessel disease
Source: Hum Brain Mapp. 2020 Feb 22;41(10):2629–41. doi: 10.1002/hbm.24967 (PMC7294060; doi:10.1002/hbm.24967)
Supplement: Supplementary file 1 — Appendix S1: Supporting information [file HBM-41-2629-s001.pdf]

Supporting Information Table S1: MRI acquisition parameters

Sequence	Feature	CADASIL (VASCAMY)	Sporadic SVD (RUN DMC – InTENse)
	Scanner	Skyra, 3 Tesla	Prisma, 3 Tesla
	Head coil	64-channels	32-channels
3D-T1	Type	MPRAGE	MP2RAGE
	TR [ms]	2500	5500
	TE [ms]	4.37	3.84
	TI [ms]	1100	700/2500
	Flip angle [˚]	7	7/4
	Voxel size [mm]	1	0.85
FLAIR	TR [ms]	5000	5000
	TE [ms]	398	394
	TI [ms]	1800	1800
	Voxel size [mm]	1	0.85
FLASH	TR [ms]	35	35
	TE [ms]	29.5	29.5
	Flip angle [˚]	15	15
	Slice thickness [mm]	2	2
	In-plane resolution [mm]	0.9 x 0.9	0.8 x 0.8
Multi-shell DWI	TR [ms]	3800	3220
	TE [ms]	104.8	74
	Flip angle [˚]	90	90
	Slice thickness [mm]	2	1.7
	In-plane resolution [mm]	2 x 2	1.7 x 1.7
	b-values [s/mm2]	200/500/1000/3000	200/500/1000/3000
	Directions (per b-value)	3/6/30/60	3/6/30/60
	b=0 [n]	10	10
	multi-band acceleration factor	3	3
Multi-band EPI	TR [ms]	700	700
	TE [ms]	39	39
	Flip angle [˚]	52	52
	Slice thickness [mm]	3	2.4
	In-plane resolution [mm]	3 x 3	2.4 x 2.4
	multi-band acceleration factor	8	8
	volumes	675	700
se-EPI field map	TR [ms]	7100	7100
	TE [ms]	66	66
	Flip angle [˚]	90	90
	Slice thickness [mm]	3	2.4
	In-plane resolution [mm]	3 x 3	2.4 x 2.4

Abbreviations: DWI, diffusion-weighted imaging; EPI, echo planar imaging; FLAIR, fluid-attenuated inversion recovery; FLASH, fast low angle shot; MP(2)RAGE, magnetization prepared (2) rapid acquisition gradient echo(es); TE, echo time; TI, inversion time; TR, repetition time.

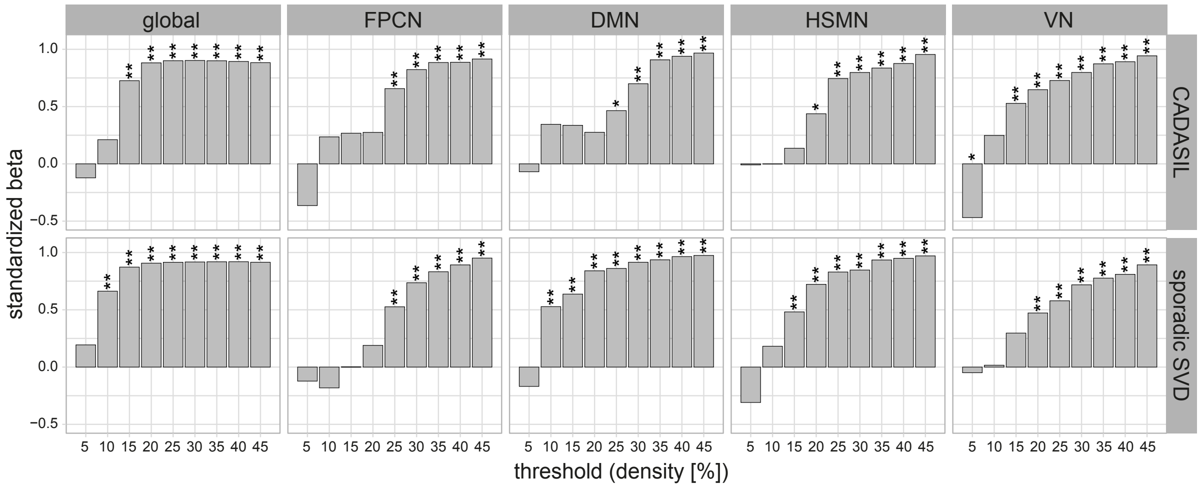

Supporting Information Figure S1 Correlations between global efficiency and clustering coefficient (* uncorrected-p < 0.01; ** uncorrected-p < 0.001)

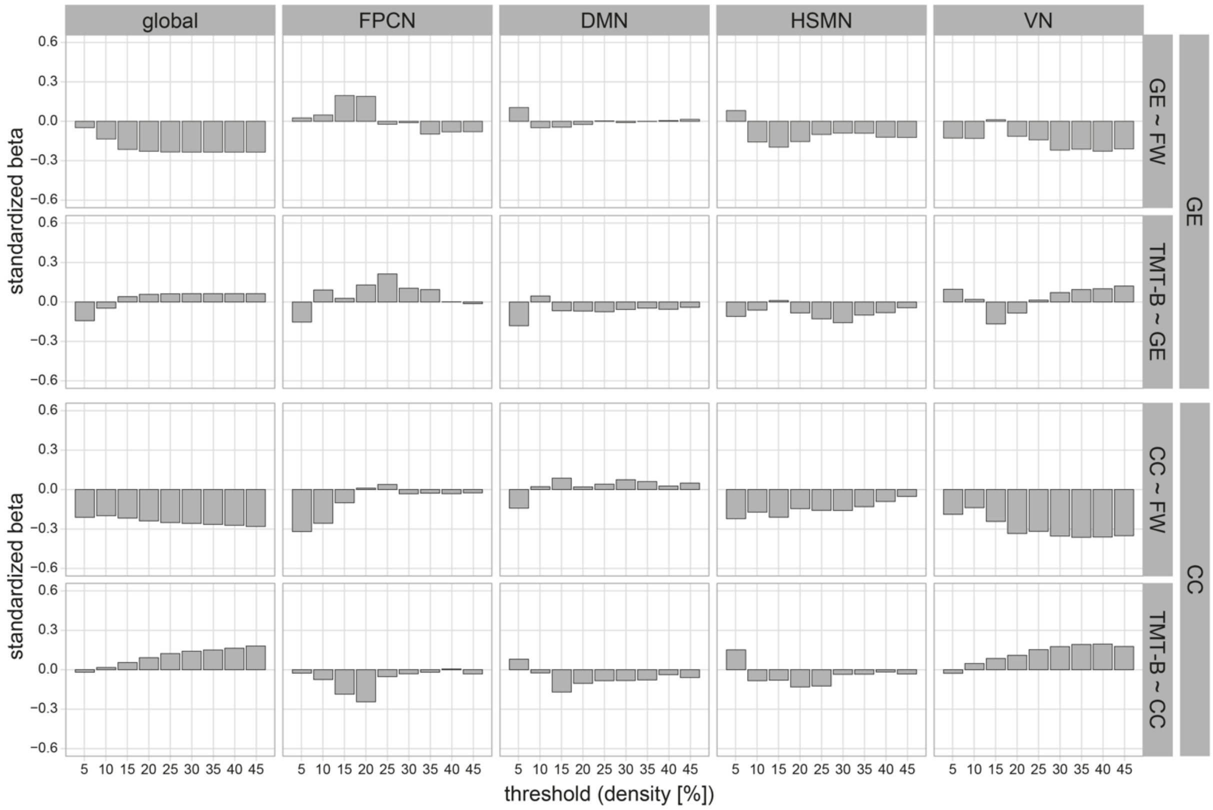

Supporting Information Figure S2 Cross-sectional analysis in sporadic SVD using simple regression. Standardized beta estimates are shown for regression of network measures against disease burden (GE ~ FW; CC ~ FW) and processing speed against network measures (TMT-B ~ GE; TMT-B ~ CC). Analyses were performed for each of the five reconstructed networks (columns) using different density thresholds (x-axis). Abbreviations: GE, weighted global efficiency; CC, weighted clustering coefficient; FW, free water content within main white matter tracts; TMT-B, trail making test matrix B; global, global network; FPCN, fronto-parietal task control network; DMN, default mode network; HSMN, hand somatosensory-motor network; VN, visual network.

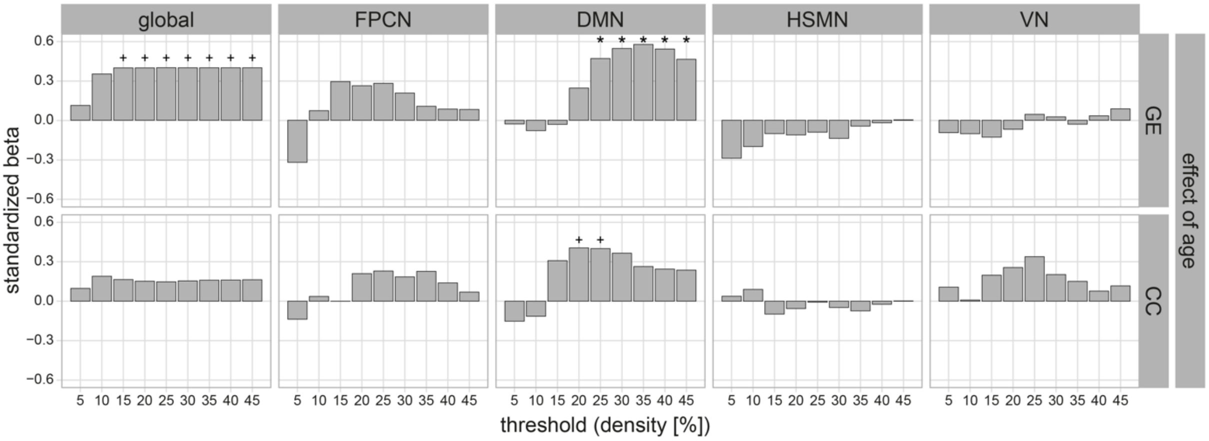

Supporting Information Figure S3 Standardized beta estimates for simple regression analyses of subject-wise calculated standard deviations of the network measures (GE, weighted global efficiency; CC, weighted clustering coefficient) against age. Analyses were done for each of the five reconstructed networks (in columns), and for different density thresholds (x-axis). * $p < 0.05$ (Bonferroni corrected). + $p < 0.1$ (marginally significant after correction). Abbreviations: global, global network; FPCN, fronto-parietal task control network; DMN, default mode network; HSMN, hand somatosensory-motor network; VN, visual network.
